# Killing filarial nematode parasites: role of treatment options and host immune response

**DOI:** 10.1186/s40249-016-0183-0

**Published:** 2016-10-03

**Authors:** Alexander Kwarteng, Samuel Terkper Ahuno, Freda Osei Akoto

**Affiliations:** 1 Kumasi Centre for Collaborative Research in Tropical Medicine (KCCR), PMB, KNUST, Kumasi, Ghana; 2 Department of Biochemistry and Biotechnology, Kwame Nkrumah University of Science Technology, PMB, Kumasi, Ghana

**Keywords:** Ivermectin, Lymphatic filariasis, Onchocerciasis, Immune response

## Abstract

**Background:**

There is compelling evidence that not only do anti-filarials significantly reduce larval forms, but that host immune responses also contribute to the clearance of filarial parasites; however, the underlying mechanisms have not been fully elucidated.

**Main text:**

Filarial infections caused by Wuchereria bancrofti and Brugia species (lymphatic filariasis) and Onchocerca volvulus (onchocerciasis) affect almost 200 million individuals worldwide and pose major public health challenges in endemic regions. Indeed, the collective disability-adjusted life years for both infections is 3.3 million. Infections with these thread-like nematodes are chronic and, although most individuals develop a regulated state, a portion develop severe forms of pathology. Mass drug administration (MDA) programmes on endemic populations focus on reducing prevalence of people with microfilariae, the worm's offspring in the blood, to less than 1 %. Although this has been successful in some areas, studies show that MDA will be required for longer than initially conceived.

**Conclusion:**

This paper highlights the mode of action of the various antifilarial treatment strategies and role of host immune response.

**Electronic supplementary material:**

The online version of this article (doi:10.1186/s40249-016-0183-0) contains supplementary material, which is available to authorized users.

## Multilingual abstracts

Please see Additional file 1 for translations of the abstract into the six official working languages of the United Nations.

## Introduction

Human filarial infections caused by nematode parasites include lymphatic filariasis (LF), onchocerciasis, loaisis and mansonellosis. These infections are believed to affect almost 200 million individuals with the major burden in developing countries [1]. The adult worm of these filarial nematodes may reside either in the lymphatic vessels or in subcutaneous tissues, and produce millions of offspring (microfilariae, MF). In LF, *W. bancrofti* infection, asymptomatic groups present two phenotypes: patent infection, which is defined by the presence of MF in peripheral blood or latent infection, characterized by the absence of MF but the presence of adult worms. Given that the latter group lack MF, they are unable to transmit the infection and essentially constitute a dead-end to the infection. Latent infection state reflects the existence of natural protective immunity in filarial endemic regions and shows distinct immune profiles to MF+ infected individuals [2]. In contrast, nearly all *O. volvulus*-infected individuals have dermal-residing MF and amicrofilaridermic patients only exist due to pre-latent infections (before the worms produce MF) or through repeated rounds of ivermectin treatment, however, both groups show distinct immune profiles [3]. In lymphatic filariasis, the pathology associated with these infections, as seen in the form of lymphedema and/or hydrocele, is caused by the presence of adult worm, whereas severe and debilitating skin disease is driven by the transmission stage (MF), in onchocercal infections. These pathologies have both economic and social consequences, including poor school performance, low productivity, low income, higher health related costs among infected adults, and a reduced life span [4].

Among the control strategies currently used in these infections include, classical antifilarial drugs in endemic regions, and vector control programmes. In addition, there has been growing evidence that public health initiatives targeted at improved water, sanitation and hygiene in endemic areas could help promote the control of vectors and the total reduction of morbidity and mortality. Despite the fact that, these strategies have achieved tremendous success in many facets, the host immune responses is also believed to contribute significantly to the clearance of the larval forms of these parasites. In this vein, the roles of current filarial treatment options and host immune response have been carefully reviewed in the sections below.

### Treatment options in lymphatic filariasis and onchocerciasis

In accordance with current mass drug administration (MDA) programs, the mainstay chemotherapy against lymphatic filariasis and onchocerciasis are combinations of ivermectin (IVM), diethlycarbamazine (DEC) with Albendazole (ALB) for LF and IVM for onchocerciasis [5]. The activity of these drugs is seen in their profound ability to kill MF, as well as late embryonic stages inside the adult female worms. However, these therapies have little effect on adult worms themselves, therefore the aim of MDA is to break transmission [4].

### Ivermectin

Ivermectin, the widely-used antifilarial drug, is a macrocyclic lactone with broad spectrum activity on filarial parasites. The drug interacts with postsynaptic glutamate-gated chloride channels (GluCl), which results in paralysis of the MFs. Interestingly, the targeted proteins (GluCl) are only encoded in the genome of Nematoda and Arthropoda, therefore restricting the effects of IVM to organisms belonging to these phyla [6]. The blocking of the GluCl channels in nematodes by ivermectin inhibits the release of uterine microfilariae by female worms and immobilizes skin and ocular microfilariae. Microfilariae are thus transported to the regional lymph nodes, where the immobilized larvae are killed by effector cells. In filarial infections, IVM exhibits profound microfilaricidal effects. However, observations in *in vitro* settings of the *Acanthocheilonema viteae* model system with IVM show a weaker activity compared to the potent killing patterns in other parasite-infected hosts [7], therefore, emphasizing the role of host immune responses in controlling the parasite. Although IVM acts primarily by rapid clearance of MF from the periphery, its role in suppressing the production of MF is remarkable. It has been hypothesized that paralysis of the pharynx of the adult female worm by IVM may lead to an absence of iron, which is a prerequisite for parasite growth and for production of MF. One important potential cause for immune responses against IVM is the genetic background of the host. Gene polymorphisms, particularly in cytokine genes associated with either immunosuppression to onchocerciasis (IL-10, TGF-β) or protection (IFN-γ, IL-4, IL-5), may contribute to varying therapeutic success of IVM in different parasite hosts. There is evidence supporting the role of IL-13 in hyper-reactive onchocerciasis [8] and TGF-β in lymphatic filariasis [9]. An overreaction (sowda) of the immune system against these worms is associated with the same variant of the IL-13 gene that confers an IgE-independent risk for asthma and atrophy [8]. Moreover, a TGF-β Single Nucleotide Polymorphism (SNP) known to be linked to reduced expression of the protein is associated with the lack of MF in the blood of patients with lymphatic filariasis [9]. Therefore, the contribution of the immune response to MF killing and genetic variation in the human population is interrelated.

### Albendazole

Albendazole (ALB) is a carbamate benzimidazole, broad-spectrum anthelminthic drug against flatworms, nematodes and cestodes that inhibits the polymerization of worm β-tubulin and microtubule formation [10]. Whether ALB has demonstrable antifilarial effects is still unclear [11]. But it has been reported to increase compliance of mass drug administration (MDA) program because of its direct effect on other gastrointestinal helminths.

### Diethlycarbamazine

Diethlycarbamazine, the piperazine derivative, attacks filarial parasites at all stages of the parasite life-cycle. However, the exact mechanism of DEC remains to be elucidated. DEC is the most effective drug for human filarial infection. Its pharmacological effects against hookworm and ascariasis, the intestinal nematode parasites, have also been proven by other studies [12]. Some studies have suggested that DEC has an indirect, host-mediated mode of action along with anti-inflammatory effects during treatments [13]. Elsewhere, DEC has been shown to inhibit the cyclooxygenase pathway (COX) and lipoxygenase pathways of parasites resulting in MF death and when administered to infected subjects results in a sharp decline in MF loads and an estimated adulticidal effect of 40 % [14]. Moreover, some studies suggest that the drug inhibits nuclear transcription factor kappa B (NF-kB) activation, a key regulator of proinflammatory genes such as TNF-α, IL-1β, as well as inducible nitric oxide synthase (iNOS) and cyclooxygenase 2 (COX-2)] [13]. In addition DEC alters the host arachidonic acid and nitric oxide metabolic pathways resulting in the immobilization and sequestration of these parasites through a yet-to-be-elucidated pathway [13, 15]. However, due to its severe adverse effects, DEC is not recommended for MDA in onchocerciasis endemic areas where it may induce local inflammation in subjects with ocular MF [5].

Because of the unique activities of each of the above mentioned drugs, specific combination therapies are used in filarial endemic regions. For instance, to treat lymphatic filariasis, IVM or DEC, in combination with ALB is used by the Global Program to Eliminate Lymphatic Filariasis (GPELF), whereas IVM is primarily used to treat onchocerciasis. Surprisingly, despite the microfilaricidal effects of these classical antifilarial drugs, they show minimal macrofilaricidal effects [16]. While IVM rapidly eliminates MF, this transmission life-stage has been reported in some endemic communities to reappear after 3 months [17], suggesting that several rounds of treatment are required to bring the MF threshold to a level below which transmission can be successfully interrupted.

### Doxycycline

Interestingly*,* the endosymbiont *Wolbachia,* has been shown to be essential for the growth and survival of most filarial worms and has therefore become the focus of alternative therapy; the application of tetracycline antibiotics from field studies has shown that 200 mg of doxycycline therapy for 4–6 weeks eliminates adult worms [18, 19]. Doxycycline is the first and, so far, only macrofilaricidal drug against onchocerciasis. Recent studies have also shown that rifampicin exhibits macrofilaricidal activities [20]. More importantly, Mand et al. have observed that doxycycline to be effective in patients without active infection since it demonstrated exceptional anti-proliferative activity leading to improved pathology conditions [21]. This suggests the use of this drug as an effective tool for individual drug treatment in filarial endemic areas [22]. While doxycycline application in field studies has shown macrofilaricidal effects compared to conventional antifilarial drugs [23], its universal application has been hampered due to contraindications among pregnant women and children under nine years. This coupled with current reports of IVM resistance in some endemic communities [24] indicates the need for the development of new and effective antifilarial drugs or vaccines if the goal to eliminate LF and onchocerciasis is to be achieved by 2020 and 2025, respectively.

### The host immune intelligence: Using all possible tactics

Whilst the activities of the various treatment options used in human filarial infections are well documented, the host immune determinants i.e., mechanisms that lead to killing huge multicellular parasites such as filarial nematodes, are currently not well defined, and remain elusive [25]. In response to an early infection of filarial nematodes, the innate defence mechanisms are initiated. Here cells such as neutrophils and eosinophils may be found around the site of infection. Larval migration leads to skin mast cells degranulations. More interestingly, filarial extracts have been shown to stimulate Toll-like receptors-dependent response [1, 26], which results in the release of inflammatory cytokines such as IL-6 and TNF-α. Of note, studies by Taylor et al. showed that *Wolbachia*-associated molecules such as *Wolbachia* surface protein (WSP) and *Wolbachia* derived protein induce innate immune response [27]. Of essence, these molecules are recognized by TLR-2,-4,-6. However, several lines of evidence points to the role of an antibody dependent cytotoxicity (ADCC) [28]. Engagement of Fc receptors (FcR) with the Fc portion of antibodies bound to helminths, ensures the recruitment of potent innate immune cells, particularly eosinophils, macrophages and neutrophils. These cells release toxic granules after activation, which result in parasite killing [29].

In addition, IFN-γ has been implicated in a *B. malayi* infection model to mediate MF killing through the release of nitric oxide [30]. It is clear that endemic normal subjects and filarial pathology patients produce increased levels of IFN-γ, hence are protected from the infective larvae. Studies in a *B. pahangi* cat model suggest that in as much as there could be a common immunological response that destroys nematode parasites, the existence of parasite stage-specific responses are of great interest to current filarial research [31]. However, this area of differential immune response apparently induced through host parasite interaction needs to be fully characterized [32].

In a highly filarial-infected individual, there can be over 50,000 MF produced on a daily basis [33]. MF have a limited life-span and their death can induce an inflammatory reaction involving the actions of neutrophils, eosinophils and macrophages. Cellular and humoral reactions to MF are usually strong in primarily infected individuals, killing the MF and often causing pathology. T helper type-2 responses are a major defence mechanism against the parasites, however, in a minority of infected individuals that develop a chronic hyper-reactive form of the infection (so-called sowda) as in the case of onchocercal infections, when these responses are not regulated. Sowda is characterized by a sustained and strong immunological defence machinery that is able to kill the MF, however at the expense of skin integrity and the individual´s well-being [8, 34]. The T helper type 2 responses are characterized by the increased production of immunoglobulins of different isotypes such as IgE [31]. Control of the parasites is associated with high IgE and IL-4 responses as well as IL-5 and eosinophilia. In individuals infected with *O. volvulus,* IL-5 is inversely correlated with the number of MF [3].

Furthermore, natural killer (NK) cells are large lymphocytes that are principally cytotoxic but have a high immunomodulatory capacity and are able to secrete mediators that influence immune responses when activated. NK cells play an important role during infection, especially toward intracellular microorganisms. Although a recent study has shown that both CD16^bright^ and CD56^dim^ as well as CD16^dim^ and CD56^bright^ NK cell populations are higher in EN when compared to individuals with generalized onchocerciasis and hyperreactive groups [32], their characterisation in other filarial infections and function requires further study. Granulocytes are generated from hematopoietic stems cells and subsequently differentiate into myeloid progenitor lineages. In fact, in circulating leucocytes of healthy humans, granulocytes consist of approximately 50 % neutrophils, whereas eosinophils and basophils make up 2–5 % and 1 %, respectively. Largely these cells are normally induced during helminth infections [35]. The role of granulocytes in filariasis appears to be diverse. They are believed to either promote protective immunity or even facilitate parasite establishment. Interestingly, eosinophils are not only associated with helminth infections but are hallmarks of allergic responses, such as in asthma and some viral infections. Peripheral eosinophil counts may reach up to 75 % during filarial infections and can induce tropical pulmonary eosinophil (TPE) in *W. bancrofti-* and *B. malayi*-infected individuals. Eosinophils contribute to the destruction of helminths by antibody-dependent cytotoxicity [36]. Activated eosinophils release granule proteins, such as ribonuclease (RNASΕ 2 and RNASE3), Eosinophil Cationic Protein (ECP), Major Basic Protein (MBP) and Eosinophil Peroxidase (EPO). Studies in EPO and MBP knockout mice have demonstrated that, in the absence of eosinophils together with its secretory granules, worms (*Litomosoides sigmodontis*) grow faster. This supports the supposition that eosinophils facilitate MF clearance during filarial infections [29]. In contrast, other studies have suggested that eosinophils are essential for early worm development [37].

Similar to eosinophils, studies in BALB/c laboratory mice have shown that neutrophils control filarial nematodes in an IL-5 dependent manner: *L. sigmodontis* infections in mice with an impaired capacity to activate neutrophils exhibited diminished parasite clearance [38]. In onchocerciasis, neutrophils are recruited to the site of infection and are influenced by the presence of *Wolbachia* endosymbionts [35]. In this study, neutrophils were found to accumulate around nodules obtained from placebo treated subjects compared to doxycycline treated counterparts. Basophils are a key cell type in the initiation of Th2 immune response since they produce IL-4. Studies in mice with *L. sigmodontis* infection showed that IL-4 is produced by basophils and that depletion of basophils resulted in drastic reduction in eosinophils and CD4^+^ T cell proliferation [39].

In addition to neutrophils, macrophages migrate to sites where filarial nematodes are located. Alternatively activated macrophage (AAM) occurs after exposure to the Th2 cytokines IL-4 and IL-13, a scenario which is normally found in late infections. These macrophages are characterised by the expression of arginase 1, the secreted chitinase-like lectin Ym-1 and resistin-like molecule. AAMs secrete cytokines, which regulate immune responses and facilitate tissue repair as well as support survival of filarial nematodes in the host via the release of IL-10 and transforming growth factor (TGF)-β consequently leading to immunosuppression. Furthermore, a recent study by Sharma and colleagues in a Tropical Pulmonary Eosinoplia (TPE) mice model showed that the pathogenesis of TPE is associated with functional impairments of alveolar macrophages, alternative activation of lung macrophages, and upregulation of antiapoptotic genes by eosinophils [40]. Since filarial infections are chronic, much research has focused on adaptive immune responses (T and B cell responses). CD4^+^ helper T cells form the majority of T lymphocyte responses and following activation differentiate into effector Th1, Th2, Th17 and regulatory T cell subsets depending on the source of antigen and cytokine milieu as reviewed in [41]. These T helper cell lineages are regulated by T-bet (Th1), GATA-3 (Th2), RORγT (Th17) and FOXP3 (Tregs), respectively. In addition, these distinct effector T cell subsets play diverse roles in mediating immune responses through the secretion of cytokines and interactions with different cell types. In LF, the immune response of MF+ individuals are characterised by T cell hypo-responsiveness which is accompanied by diminished production of IFN-γ and IL-2 [42].

More recently, studies in onchocerciasis showed a strong association of Th2 and Th17 responses in individuals presenting hyper-reactive onchocerciasis (HO) [32]. In that study, HO patients presented a reduced regulatory phenotype when compared to generalised onchocerciasis individuals. The study also revealed that in comparison to infected individuals, EN exhibited a pronounced Th1 phenotype since the frequency of IFN-γ producing CD4^+^ T cells and released IFN-γ upon filarial-specific re-stimulation of PBMCs were both elevated. Such observations indicate that Th1 responses contribute to parasite control.

## Conclusion

This review presents the various forms of parasite control during human filarial infections with a focus on treatment options and role of the host immune response. The information presented in this review may not have discussed all the mechanisms involved, but it has explained some of the activities of antifilarial drugs while underscoring the roles of immune components, understood to be instrumental in killing nematode parasites during infection.

## Additional file

Additional file 1: Multilingual abstracts in the six official working languages of the United Nations. (PDF 695 kb)

## Abbreviations

ALB: Albendazole
DEC: Diethlycarbamazine
IVM: Ivermectin
LF: Lymphatic filariasis
MDA: Mass drug administration
MF: Microfilariae
TPE: Tropical pulmonary eosinophilia
