# Supplementary material for: Killing filarial nematode parasites: role of treatment options and host immune response
Source: Infect Dis Poverty. 2016 Oct 3;5:86. doi: 10.1186/s40249-016-0183-0 (PMC5047298; doi:10.1186/s40249-016-0183-0)
Supplement: Additional file 1: — Multilingual abstracts in the six official working languages of the United Nations. (PDF 695 kb) [file 40249_2016_183_MOESM1_ESM.pdf]

Translation of the abstract into the six official working languages of the United Nations

قتل طفيليات فيلاريا الخيطية: دور خيارات العلاج و الاستجابة المناعية للمضيف

الكساندر كوارتنج، ساموبل تيركبير اهونو و فريدا اوسي اكوتو

ملخص

خلفية: هناك أدلة دامغة على أن الفيلاريات لا تفعل فقط تقليل أشكال اليرقات إلى حد كبير ، ولكن أن الاستجابة المناعية أيضا أن تسهم في إزالة طفيليات خيطية. ومع ذلك، فإن الآليات الكامنة لم توضح تماما.

النص الرئيسي: التهابات الفيلاريا الناجمة عن فخرية بنكروفتية والأنواع البروجية (داء الخيطيات اللمفاوية) وكلابية الذنب المتلوية (داء كلابية الذنب) تؤثر على ما يقرب من 200 مليون شخص في جميع أنحاء العالم وتطرح تحديات صحية عامة رئيسية في المناطق الموبوءة. والواقع أن سنوات العمر المصححة باحتساب مدد العجز الجماعية هي 3.3 مليون. العدوى مع هذه الديدان الخيطية مزمنة، وعلى الرغم من أن معظم الأفراد يطوروا حالة منظمة، جزء منهم يطور أشكال حادة من الأمراض. وتركز البرامج إدارة المخدرات (MDA) الجماعية على السكان المستشري على الحد من انتشار الناس مع الميكروفلاريا، نسل الدودة في الدم، إلى أقل من 1٪. على الرغم من أن هذا كان ناجحا في بعض المناطق، تظهر الدراسات أنه ستكون هناك MDA لمدة أطول من تصور في البداية.

استنتاج: هذه الورقة يتسلط الضوء على طريقة عمل مختلف من استراتيجيات علاج مضاد الفيلاريا ودور الاستجابة المناعية للمضيف.

Translated from English version into Arabic by Bamo A. Aziz, through

TRANSLATORS
WITHOUT BORDERS

杀灭丝虫：治疗方案选择和宿主免疫反应的作用

Alexander Kwarteng, Samuel Terkper Ahuno and Freda Osei Akoto

摘要

背景：有力证据表明，抗丝虫不仅能显著减少丝虫幼虫数量，而且宿主免疫反应也有助于丝虫的清除。然而，其内在机制尚未完全阐明。

主要内容：班氏丝虫（*Wuchereria bancrofti*）和布鲁丝虫属（*Brugia*）引起的淋巴丝虫病，以及旋盘尾丝虫（*Onchocerca volvulus*）引起的盘尾丝虫病威胁全球近 2 亿人的健康，是流行区重大的公共卫生挑战。事实上，两种疾病共同导致了 330 万年的残疾调整生命年的损失。上述两种疾病以慢性感染为主，尽管大多数患者能达到控制的状态，但一部分人会发展为严重病理状态。针对丝虫病流行区人群的群体预防服药(MDA)项目专注于减少感染人群血液微丝蚴含量（1%以下）。虽然 MDA 项目在某些区域取得成功，但多项研究表明 MDA 需要比最初设想的时间更长。

结论：本文突出论述多种抗丝虫治疗策略的实施方法和宿主免疫反应的作用。

Translated from English version into Chinese by Hong Tu, edited by Pin Yang

CDC CHINESE CENTER FOR DISEASE CONTROL AND PREVENTION
NATIONAL INSTITUTE OF PARASITIC DISEASES

Éliminer les nématodes parasites : rôle des traitements possibles et de la réponse immunitaire de l’hôte dans la lutte contre la filariose

Alexander Kwarteng, Samuel Terkper Ahuno et Freda Osei Akoto

Résumé

Contexte: Il semble aujourd'hui bien démontré que les antifilariens réduisent significativement les formes larvaires des filaires, mais aussi que les réponses immunitaires de l'hôte contribuent à l'élimination de ces parasites. Les mécanismes de ces effets ne sont cependant pas entièrement connus.

Corps de texte: Les filarioses causées, d'une part, par *Wuchereria bancrofti* et *Brugia* sp. (filarioses lymphatiques) et, d'autre part, par *Onchocerca volvulus* (onchocercose) affectent presque 200 millions de personnes dans le monde et posent de sérieux problème de santé publique dans les régions d'endémie. Ces deux parasitoses représentent en effet un poids de 3,3 millions d'années d'espérance de vie collective corrigée du handicap. Les infestations par ces nématodes filiformes causent des maladies chroniques, qui peuvent s'établir à un stade régulé dans la plupart des cas mais évoluent parfois vers des formes sévères. Les programmes de traitement médicamenteux de masse (TMM) à destination des populations des zones d'endémie visent principalement la réduction à moins de 1 % de la prévalence de infestations par les microfilaires (la progéniture des vers en circulation dans le sang). Même si cet objectif a été atteint dans certaines zones, les études montrent que le TMM devra durer plus longtemps qu'initialement prévu.

Conclusion: Le présent article met en évidence le mode d'action des différentes stratégies de traitement antifilarien et le rôle de la réponse immunitaire de l'hôte.

Translated from English version into French by Suzanne Assenat, through

TRANSLATORS WITHOUT BORDERS

Уничтожение филярийных паразитных нематод: Роль вариантов лечения и иммунного ответа на восприимчивость

Александр Квартенг, Самюэль Теркпер Ахуно и Фреда Осей Акото

Аннотация

История: Существуют убедительные доказательства того, что не только анти-филярийные препараты значительно уменьшают личиночные формы, но и иммунный ответ на восприимчивость также способствуют клиренсу филярийных паразитов. Тем не менее, основные механизмы пока не полностью выяснены.

Основной текст: Филяриатоз, вызванный Нитчаткой Банкрофта (вухерериоз) и возбудителями Brugia (лимфатический филяриатоз) и Onchocerca (онхоцеркоз) затрагивает почти 200 миллионов человек во всем мире и создает серьезные проблемы в области общественного здравоохранения в эндемичных районах. Действительно, срок жизни обеих инфекций с поправкой на инвалидность составляет 3,3 миллиона лет. Инфекции с этими нитевидными нематодами являются хроническими, и, хотя большинство людей приспосабливаются к существованию, у части развиваются тяжелые формы патологии. Массовые программы введения препарата (MDA) эндемическим населениям сосредоточены на снижении числа людей с микрофилярием, потомством червя в крови, менее чем на 1%. Хотя это было успешным в

некоторых областях, исследования показывают, что MDA будет требоваться дольше, чем считалось изначально.

Заключение: В настоящем документе рассматриваются различные стратегии лечения филяриатоза и роли иммунного ответа на восприимчивость.

Translated from English version into Russian by olessiala, through

TRANSLATORS
WITHOUT BORDERS

Eliminación de parásitos nematodos tipo filarias: el papel de los distintos tratamientos y la repuesta inmunológica del huésped

Autores: Alexander Kwarteng, Samuel Terkper Ahuno y Freda Osei Akoto

Sinopsis

Contexto: Existen pruebas convincentes de que no solo los antifilarios reducen notablemente el número de larvas, sino que la respuesta inmunológica del huésped también contribuye a la eliminación de este tipo de parásitos. Sin embargo, los mecanismos subyacentes no se han esclarecido por completo.

Texto principal: Las infecciones filarias provocadas por las especies *Wuchereria bancrofti* y *Brugia* (filariasis linfática) y *Onchocerca volvulus* (oncocerciasis) afectan a casi 200 millones de personas en todo el mundo y presentan una importante amenaza para la salud pública en las zonas endémicas. De hecho, ambos tipos de infección suman un total de años de vida ajustados por discapacidad de 3,3 millones. Las infecciones producidas por estos nematodos son crónicas y aunque el estado de la mayoría de los afectados suele estabilizarse, algunos desarrollan formas de patología severas. Los programas de administración masiva de medicamentos (MDA por sus siglas en inglés) a los que se somete a la población endémica se centran en reducir la prevalencia de individuos con microfilarias (larvas del gusano en la sangre) a menos del 1 %. Pese a que dicha medida ha tenido éxito en algunas zonas, los estudios revelan que los programas de MDA serán necesarios durante más tiempo del que inicialmente se preveía.

Conclusión: Este artículo se centra en la forma en que actúan los distintos tratamientos antifilarias y el papel de la respuesta inmunológica del huésped.

Translated from English version into Spanish by Marta Callava Linares, through

TRANSLATORS
WITHOUT BORDERS
